# Methyl Jasmonate Alleviated the Adverse Effects of Cadmium Stress in Pea (*Pisum sativum* L.): A Nexus of Photosystem II Activity and Dynamics of Redox Balance

**DOI:** 10.3389/fpls.2022.860664

**Published:** 2022-03-24

**Authors:** Hamid Manzoor, Mehwish, Sherien Bukhat, Sumaira Rasul, Muhammad Ishaq Asif Rehmani, Sibgha Noreen, Habib-ur-Rehman Athar, Zafar Ullah Zafar, Milan Skalicky, Walid Soufan, Marian Brestic, Muhammad Habib-ur-Rahman, Chukwuma C. Ogbaga, Ayman EL Sabagh

**Affiliations:** ^1^Institute of Molecular Biology and Biotechnology, Bahauddin Zakariya University, Multan, Pakistan; ^2^Department of Agronomy, Ghazi University, Dera Ghazi Khan, Pakistan; ^3^Institute of Pure and Applied Biology, Bahauddin Zakariya University, Multan, Pakistan; ^4^Department of Botany and Plant Physiology, Faculty of Agrobiology, Food, and Natural Resources, Czech University of Life Sciences Prague, Prague, Czechia; ^5^Plant Production Department, College of Food and Agriculture Sciences, King Saud University, Riyadh, Saudi Arabia; ^6^Laboratory Slovak University of Agriculture in Nitradisabled, Nitra, Slovakia; ^7^Crop Science, Institute of Crop Science and Resource Conservation (INRES), University of Bonn, Bonn, Germany; ^8^Department of Biological Sciences, Nile University of Nigeria, Abuja, Nigeria; ^9^Department of Agronomy, Faculty of Agriculture, Kafrelsheikh University, Kafr El-Shaikh, Egypt

**Keywords:** antioxidants, cadmium toxicity, methyl jasmonate, oxidative stress, photosystem II

## Abstract

The accumulation of cadmium (Cd) in leaves reduces photosynthetic capacity by degrading photosynthetic pigments, reducing photosystem II activity, and producing reactive oxygen species (ROS). Though it was demonstrated that the application of Methyl Jasmonate (MeJA) induces heavy metal (HM) stress tolerance in plants, its role in adjusting redox balance and photosynthetic machinery is unclear. In this study, the role of MeJA in modulating photosystem II (PSII) activity and antioxidant defense system was investigated to reduce the toxic effects of Cd on the growth of pea (*Pisum sativum* L.) cultivars. One-week-old seedlings of three pea varieties were subjected to Cd stress (0, 50, 100 μm), and MeJA (0, 1, 5, 10 μm) was applied as a foliar spray for 2 weeks. Cadmium stress reduced the growth of all three pea varieties. Cadmium stress decreased photosynthetic pigments [Chl a (58.15%), Chl b (48.97%), total Chl (51.9%) and carotenoids (44.01%)] and efficiency of photosystem II [Fv/Fm (19.52%) and Y(II; 67.67%)], while it substantially increased Cd accumulation along with an increase in ROS (79.09%) and lipid peroxidation (129.28%). However, such adverse effects of Cd stress varied in different pea varieties. Exogenous application of MeJA increased the activity of a battery of antioxidant enzymes [superoxide dismutase (33.68%), peroxidase (29.75%), and catalase (38.86%)], improved photosynthetic pigments and PSII efficiency. This led to improved growth of pea varieties under Cd stress, such as increased fresh and dry weights of shoots and roots. In addition, improvement in root biomass by MeJA was more significant than that of shoot biomass. Thus, the mitigating effect of MeJA was attributed to its role in cellular redox balance and photosynthetic machinery of pea plants when exposed to Cd stress.

## Introduction

The excessive uptake and accumulation of heavy metals, including cadmium (Cd), inhibit plants’ growth and development (Kranner and Colville, 2011; Haider et al., 2022). The presence of cadmium disturbs plant functions, such as uptake of water and mineral nutrients inhibition of photosynthetic machinery (Baryla et al., 2001). In addition, Cd stress causes the over a reduction of NADPH and thus cause an imbalance in electron transport from photosystem II (PSII) to photosystem I (PSI) and consumption of electrons in generating reducing equivalents, thereby resulting in ROS (reactive oxygen species) production. The generated ROS species at the PSI end can cause photoinhibition of PSII and PSI. Plants can avoid PSI photoinhibition by limiting the electron transport from PSII to PSI (lowering PSII activity) or re-routing electrons by providing alternative electron acceptors, such as cyclic electron transport (Taiz et al., 2015; Ogbaga and Athar, 2019; Shahzadi et al., 2021; Umer Chattha et al., 2021). These antioxidant enzymes scavenge ROS and protect the cellular structures (Bukhat et al., 2020; Askari-Khorasgani et al., 2021; Yasir et al., 2021). Photosystem II is more protected from ROS species than PSII by a battery of antioxidant enzymes (Taiz et al., 2015; Farid et al., 2018; Tikkanen and Grebe, 2018), which include superoxide dismutase (SOD), ascorbate peroxidase (APX), and catalase (CAT; Foyer et al., 2012; Foyer, 2018; Nazir et al., 2021). However, photoinhibition of PSII is protected by activating the xanthophyll cycle photoprotective component of non-photochemical quenching (NPQ). Sufficient evidence is available that demonstrates that various plant growth regulators, osmoprotectants, antioxidant signaling compounds can efficiently modulate nutrient uptake and transport, PSII activity, and antioxidants, thereby regulating plant growth under normal or stress conditions (Zhao et al., 2013; Athar et al., 2015; Ahmad et al., 2019; Ayyaz et al., 2021).

Jasmonate (JA) and methyl jasmonate (MeJA; methyl ester of JA) are well-known plant growth regulators that affect different biochemical and physiological processes (Yu et al., 2019; Bukhat et al., 2021), such as stomatal opening and photosynthetic activity (Yan et al., 2015). Several studies have shown that MeJA induces a stimulatory effect on photosystem II (PSII) photochemistry and photosynthetic pigments under normal conditions (Attaran et al., 2014; Qiu et al., 2020). While working with *Brassica juncea* Per et al. (2016) evidenced from transmission electron microscopy that MeJA protected the structure of chloroplast from cadmium toxicity. In addition, some studies showed that MeJA modulates the activities of some key antioxidant enzymes in different plants during heavy metal stress, such as in *B. juncea* (Per et al., 2016), Kandelia obovata (Chen et al., 2014), and *Brassica napus* (Farooq et al., 2018) Likewise, several studies showed that MeJA application in lower concentrations improved plant tolerance against abiotic stresses including Cd stress (Walia et al., 2007; Keramat et al., 2009; Per et al., 2016).

Pea (*Pisum sativum* L.) is one of the most crucial leguminous vegetable crop, whose yield is affected by Cd stress. Because of available information about MeJA, it is hypothesized that MeJA application might have improved the growth and yield of pea under cadmium stress. Although it is known that MeJA can change the PSII activity and activities of antioxidant enzymes, it is not yet known whether MeJA can also modulate solar energy absorption by PSII, and its distribution in electron transport or photochemistry and xanthophyll cycle under heavy metal stress conditions, including Cd stress. Since MeJA suppresses the growth and photosynthetic activity under stress conditions, when jasmonate signaling activates antioxidants enzymes and redirects metabolism from growth to defense, it is apt to assess up to what extent exogenously applied MeJA modulates the antioxidant mechanism and photosynthetic responses in pea plants to alleviate the adverse effects of Cd stress. The study’s secondary objective was to assess genotypic variability for these responses in local pea germplasm.

## Materials and Methods

### Plant Materials and Treatments

A pot experiment was conducted in a randomized block design with three local peas (*Pisum sativum* L.) cultivars (Meteor, S-Green, and Climax), four levels of cadmium (Cd) stress, and three levels of methyl jasmonate (MeJA) as a foliar spray with four replicates. Seeds of three pea cultivars were obtained from Ayub Agricultural Research Institute (AARI), Faisalabad, Pakistan. Seeds were disinfected with sodium hypochlorite before sowing. The experiment was conducted under controlled conditions (Light/Dark period 12/12 h, Humidity: 60%, Light intensity: 180–190 μmol m^−2^ s^−1^ and 20–25°C temperature) in growth room at the Institute of Molecular Biology and Biotechnology, Bahauddin Zakariya University, Multan, Pakistan. Pea seeds were sown in plastic pots filled with a mixture of sand and soil (3:1). Germinating seeds were supplemented with Hoagland nutrient solution. After 2 weeks of germination, healthy and homogenous plants were selected. Pea plants of the three cultivars were treated with different concentrations (0, 1, and 10 μm, foliar spray) of MeJA containing 0.01% Tween-20. Subsequently (72 h after MeJA treatment), plants were exposed to different levels of cadmium stress (0, 50, 100, and 200 μm CdCl_2_). Cadmium stress was given from 50 μm and gradually increased to attain the required concentrations. Physiological and biochemical parameters were measured 48 h after the last cadmium treatment in four biological replicates.

### Measurement of Biomass

After the completion of the duration of cadmium stress, pea plants were uprooted carefully, and plant parts were separated into shoots and roots. Plant parts were blotted dry, and their fresh weights (g) of shoots and roots were measured using a digital scale. Dry weights pg. shoots and roots of all the three cultivars of pea were recorded after drying the samples in the oven at 65°C for 1 week. The root length (cm) of each cultivar was measured using a ruler.

#### Determination of Photosynthetic Pigments and Photosystem II Photochemistry

Photosynthetic pigments were extracted from 0.5 g of fresh leaves samples using 80% acetone by placing them in the dark for 24 h. The absorbance of the extracted photosynthetic pigments (Chlorophyll a, b, total chlorophyll, and carotenoids) was measured at 480 nm, 663 nm, and 645 nm using a double beam spectrophotometer. The number of photosynthetic pigments was calculated using formulae as described elsewhere (Wildermuth and Fall, 1996). The maximum quantum yield of photosystem II (PSII) and distribution of absorbed solar energy in driving photochemistry and in processes other than photochemistry, such as a photoprotective component of non-photochemical quenching (NPQ), photoinhibition of PSII were evaluated using chlorophyll fluorescence induction analysis with DUAL-PAM 100 (Walz, Effeltrich, Germany). Briefly, plants were dark-adapted for 30 min, and then initial and maximum fluorescence (Fo, Fm) were investigated by applying week light (0.3 μmol photons/m^2^/s^1^ and then saturating light pulse of 0.8-s; 6,000 μmol photons/m2/s1). The steady-state PSII fluorescence yield was measured by exposing the leaf to red actinic light (635 nm). The actual efficiency of PSII, electron transport rate through PSII, non-photochemical quenching (NPQ), the fraction of photoprotective component of NPQ (Y-NPQ), and a fraction of NPQ due to photoinhibition of PSII were calculated following Schreiber and Klughammer (2008).

#### Determination of Lipid Peroxidation

Lipid peroxidation was measured to estimate the damaging effects of Cd in the cellular membrane. Approximately 0.25 g of fresh leaves were homogenized in 8 ml of 0.1% trichloroacetic acid (TCA) and was centrifuged for 30 min at 16000 rpm. After centrifugation, the supernatant was mixed with TBA (3 ml) prepared in a 20% solution of TCA. This mixture was placed at 95°C for 1 h in a water bath the following cooling on ice for almost 4–5 min. The absorbance of the mixture was taken at 600 nm with a double beam spectrophotometer (PerkinElmer Ltd., United Kingdome). MDA content was measured by an extinction coefficient of 155 mm^−1^ cm^−1^ (Dhindsa et al., 1981).

#### Estimation of Reactive Oxygen Species (H_2_O_2_)

Fresh leaves (0.25 g) were homogenized in a 3 ml solution of 0.1% TCA and centrifuged at 9000 × *g* for 15 min. The supernatant (0.1 ml) was mixed with potassium iodide (0.2 ml) and 50 mm potassium phosphate buffer (0.1 ml). The mixture was vortexed, and its optical density (OD) was measured at 390 nm (Velikova et al., 2000).

#### Determination of Antioxidant Activity

Enzymatic antioxidant activity was evaluated following the procedure described by Ananieva et al. (2004). Enzyme extract was prepared by homogenizing 0.25 g fresh leaves in 2 ml of 50 mm potassium phosphate buffer (pH = 7.8), which was then centrifuged for 20 min at 15000 × *g*. The supernatant was used as enzyme extract for assessing activities of catalase (CAT), superoxide dismutase (SOD), and peroxidase (POD). CAT activity was measured by adding 0.1 ml H_2_O_2_ (300 mm), 3 ml phosphate buffer (pH = 7), and 0.1 ml enzyme extract in the reaction mixture (Miller and Rice-Evans, 1996). The samples were vortexed, and absorbance was measured at 240 nm for 1 min after 20 s. CAT activity was measured using the following formula: CAT activity = (activity^*^A ^*^ V/a)/(E·W). The reaction mixture for measuring SOD activity contained 75 μm nitroblue tetrazolium (NBT), 100 μm EDTA, 20 μm riboflavin, and 130 mm methionine and was placed in light for 1 h. After an hour, the color of the reaction mixture changed gradually, and this mixture was placed in the dark for 10 min to stop the reaction. The absorbance was taken at a wavelength of 560 nm, and its activity was evaluated using the standard curve of known concentrations of NBT (Armstrong, 1998). For POD activity, 300 mm H_2_O_2_ (0. 1 ml), 1.5% guaiacol (0.1 ml), enzyme extract (0.1 ml) and 50 mm potassium phosphate buffer (2.7 ml) were mixed. Its absorbance was measured for 2 min at 470 nm using a spectrophotometer (PerkinElmer Ltd., United Kingdome; Zhou and Leul, 1999).

#### Cadmium Quantification

Dried leaves (0.2 g) were digested in 5 ml of sulfuric acid overnight for cadmium determination. The samples were kept at 240°C on a hot plate until boiling and diluted with perchloric acid and nitric acid (1:5). This mixture was kept on a hot plate until it became transparent. Cd in this digested solution was quantified through atomic absorption spectrophotometer (240FS AA, Agilent Technologies, United States; Jackson, 2005).

### Statistical Analysis

For statistical analysis, a three-way ANOVA (completely randomized block design) was performed using COSTATv.6.451 software (CoHort Software, California, United States). The least significant difference (LSD) was used for comparing the means of the different treatments if the interaction term was found significant.

## Results

### Plant Growth

**Figure 1:**
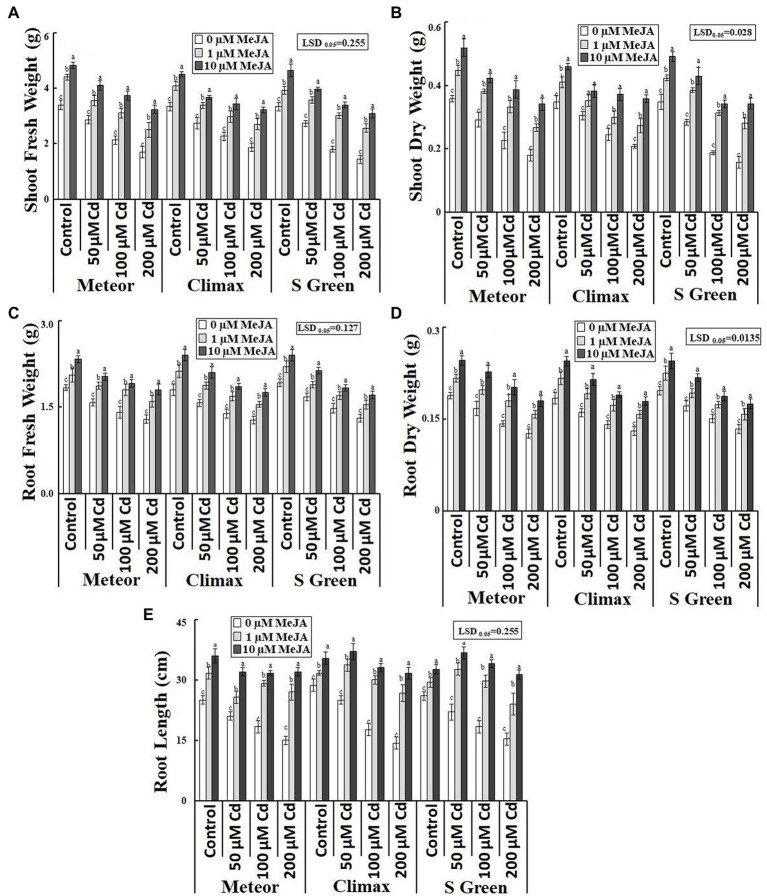
Effect of CdCl2 (50, 100, and 200 μm) and MeJA (1 and 10 μm) on **(A)** Shoot fresh weight; **(B)** Shoot dry weight; **(C)** Root fresh weight; **(D)** Root dry weight; and **(E)** Root length of three pea cultivars. Bars show a mean of 3 three replicates ± standard deviation. Letters a, b, and c showed significant differences between means of different treatments.

**Figure 2:**
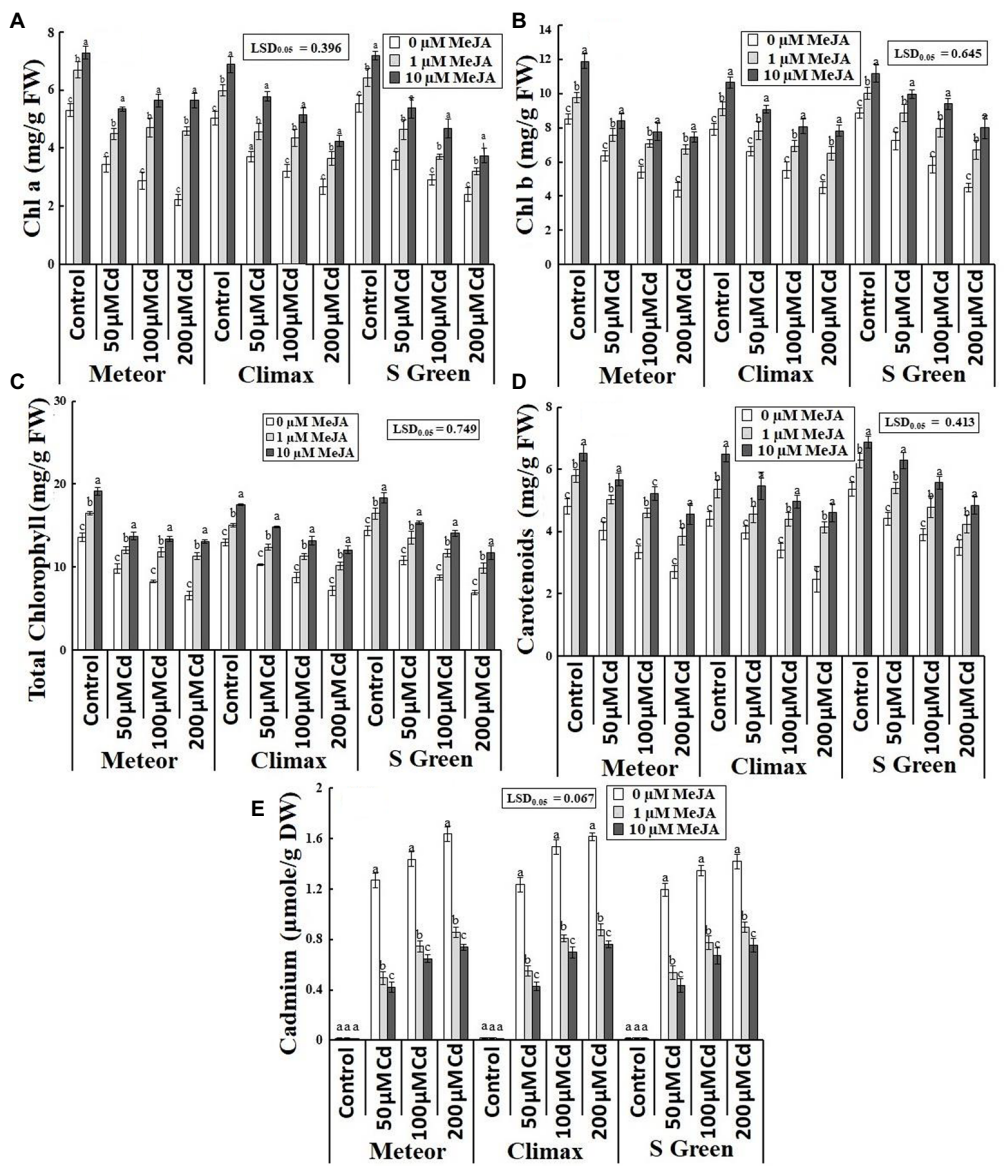
Effect of CdCl2 (50, 100, and 200 μm) and MeJA (1 and 10 μm) on **(A)** Chl a; **(B)** Chl b; **(C)** Total Chl; **(D)** Carotenoids; and **(E)** Cadmium content of three pea cultivars. Bars show a mean of 3 three replicates ± standard deviation. Letters a, b, and c showed significant differences between means of different treatments.

Increasing CdCl_2_ (50, 100, and 200 μm) stress considerably declined both fresh and dry weight of roots and shoots of all the three pea cultivars. Foliar application of MeJA (1 and 10 μm) significantly increased the biomass of the three cultivars under normal and Cd-stressed conditions (Table 1). However, the application of 10 μm MeJA was more effective in improving the biomass of the three cultivars in Cd-stressed conditions (Figures 1A–D). In addition, this increasing effect of MeJA significantly varied in three pea cultivars. For example, at 10 μm MeJA application, the cultivar S-green showed a maximum increase in shoot fresh and dry weights by 115.16 and 118.86%, respectively, at the highest Cd stress. In contrast, cultivar Climax showed a minimum increase of 72.32 and 71.93%, respectively. The cultivar Meteor showed a maximum increase in root fresh and dry weight by 39.79 and 42.61%, respectively. Similarly, 10 μm MeJA resulted in the maximum increase in root length and was observed at 200 μm CdCl_2_ in Climax (120.93%; Figure 1E). Moreover, Cd content was found to be significantly higher in Cd-stressed plants compared to the control plants. However, applying both concentrations of MeJA remarkably reduced Cd accumulation in all Cd-stressed pea cultivars (Table 1). In addition, the supplementation of 10 μm MeJA remarkably reduced Cd accumulation in all cultivars under varying concentrations of CdCl_2_ stress (50, 100, and 200 μm), particularly in Meteor by 66.57, 54.83, and 54.79%, respectively (Figure 2E).

**Table 1:** Mean square values from ANOVA for root length, root & shoot fresh & dry weight, photosynthetic pigments, MDA, H_2_O_2_, antioxidant enzyme activities and PSII photochemistry of three cultivars of pea (*Pisum sativum* L.) plant treated with different methyl jasmonate concentrations under normal and Cd-stressed conditions.

Source of variation	*df*	Shoot fresh wt.	Shoot dry wt.	Root fresh wt.	Root dry wt.	Root length
Cd	3	12.481^***^	0.126^***^	1.711^***^	0.020^***^	230.261^***^
MeJA	2	16.760^***^	0.184^***^	2.068^***^	0.023^***^	1591.08^***^
Varieties	2	0.287^***^	0.002^**^	0.011 ns	0.000175 ns	25.974^***^
Cd^*^MeJA	6	0.069^*^	0.001^**^	0.005 ^*^	0.0000553^*^	38.881^***^
Cd^*^Varieties	6	0.100^**^	0.001^***^	0.013 ns	0.0000759 ns	23.937^***^
MeJA^*^Varieties	4	0.094^**^	0.002^***^	0.008 ns	0.0001706 ns	0.856 ns
Cd^*^MeJA^*^Varieties	12	0.028 ns	0.00031 ns	0.002 ns	0.0000151 ns	5.634^**^
Error	72	0.02456	0.000308	0.00618	0.000069	2.0625
**Source of variation**	***df***	**Chl a**	**Chl b**	**Total Chl**	**Carotenoids**	**Cd Conc.**
Cd	3	35.617^***^	59.604^***^	184.819^***^	17.382^***^	6.001^***^
MeJA	2	36.492^***^	73.027^***^	214.793^***^	27.415^***^	3.722^***^
Varieties	2	1.529^***^	4.914^***^	2.421^***^	3.528^***^	0.014^***^
Cd^*^MeJA	6	0.101^*^	0.601^**^	0.919^***^	0.050^*^	0.413^***^
Cd^*^Varieties	6	0.890^***^	1.174^***^	2.727^***^	0.067 ns	0.004^*^
MeJA^*^Varieties	4	0.778^***^	0.138 ns	0.770^**^	0.053 ns	0.016^***^
Cd^*^MeJA^*^Varieties	12	0.222^***^	0.222 ns	0.394 ns	0.104 ns	0.003^*^
Error	72	0.05913	0.15738	0.21180	0.06454	0.00169
**Source of variation**	***df***	**ROS**	**MDA**	**POD**	**SOD**	**CAT**
Cd	3	0.201^***^	1140.435^***^	338.043^***^	628.65^***^	909.800^***^
MeJA	2	0.158^***^	9127.196^***^	50.177^***^	153.596^***^	113.147^***^
Varieties	2	0.059^***^	414.038^***^	37.459^***^	21.595^***^	133.714^***^
Cd^*^MeJA	6	0.00047^*^	782.336^***^	0.401^*^	0.589^*^	0.355^*^
Cd^*^Varieties	6	0.001^*^	95.114^***^	1.663^***^	3.112^**^	14.120^***^
MeJA^*^Varieties	4	0.001^**^	33.013^**^	0.179 ns	1.367 ns	1.538^**^
Cd^*^MeJA^*^ Varieties	12	0.000286 ns	23.228^**^	0.319 ns	0.367 ns	0.704 ns
Error	72	0.000449	8.58802	0.23986	0.74955	0.42484
**Source of variation**	***df***	**Fv/Fm**	**Y (II)**	**ETR (II)**	**Y (NPQ)**	**Y (NO)**
Cd	3	0.089^***^	0.283^***^	408.960^***^	0.414^***^	1.071^***^
MeJA	2	0.061^***^	0.295^***^	372.751^***^	0.095^***^	0.269^***^
Varieties	2	0.0043^***^	0.033^***^	40.230^***^	0.00048 ns	0.0010 ns
Cd^*^MeJA	6	0.00017^*^	0.00041^*^	3.747^**^	0.004^***^	0.0023^*^
Cd^*^Varieties	6	0.00015 ns	0.006^***^	1.631 ns	0.009^***^	0.0053^**^
MeJA^*^Varieties	4	0.00043 ns	0.003^**^	2.231 ns	0.00025 ns	0.0015 ns
Cd^*^MeJA^*^Varieties	12	0.000039 ns	0.00023 ns	0.617 ns	0.000368 ns	0.0013 ns
Error	72	0.00027	0.000735	0.93815	0.000496	0.00156

MeJA: Methyl Jasmonate; ^*^, ^**^ and ^***^ show significance level at 0.05, 0.01 and 0.001, respectively, while ns = non-significant.

Cd toxicity significantly reduced the photosynthetic pigments of all three pea cultivars under cadmium stress, while the MeJA application quite recovered photosynthetic pigments compared to typical and Cd-stressed plants (Table 1). Under the highest Cd stress, the supplementation of 10 μm MeJA effectively increased chl a, chl b, total chl and carotenoids in Meteor (153.92%), S-green (77.63%), Meteor (98.64%), and Climax (86.78%), respectively (Figures 2A–D).

**Figure 3:**
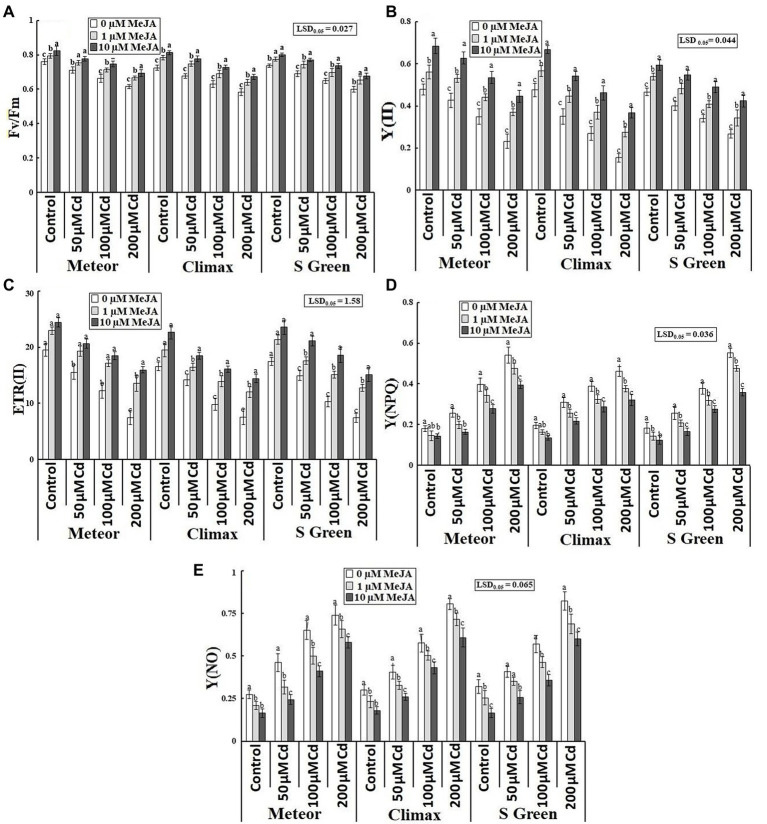
Effect of CdCl2 (50, 100, and 200 μm) and MeJA (1 and 10 μm) on **(A)** Fv/Fm; **(B)** Y(II); **(C)** ETR(II); **(D)** Y(NPQ); and **(E)** Y(NO) of three pea cultivars. Bars show a mean of 3 three replicates ± standard deviation. Letters a, b, and c showed significant differences between means of different treatments.

Cd-induced considerable reduction in electron transport rate (ETRII) and the quantum yield of PSII [in terms of Fv/Fm and Y(II)] was improved by the application of MeJA (1 and 10 μm) under normal and Cd-stressed conditions (Table 1; Figures 3A–C). The more pronounced improvement in Fv/Fm, Y(II), and ETR(II) was observed by 10 μm MeJA at the highest cadmium stress in Climax (15.32, 139.6%) and Meteor (113.05%), respectively, compared to their respective control plants. Moreover, the quantum yield of non-photoprotective and photoprotective energy dissipation [Y(NO) and Y(NPQ)] was considerably increased with increasing cadmium concentrations in all pea plants. Although, the damage caused to PSII machinery by Cd toxicity was reduced by MeJA application in all pea cultivars compared to their respective controls (Table 1). From Figures 3D,E, it can be observed that at the highest cadmium stress, the supplementation of 10 μm MeJA considerably alleviated Y(NPQ) and Y(NO) parameters in S-green by 35.25 and 26.99%, respectively.

**Figure 4:**
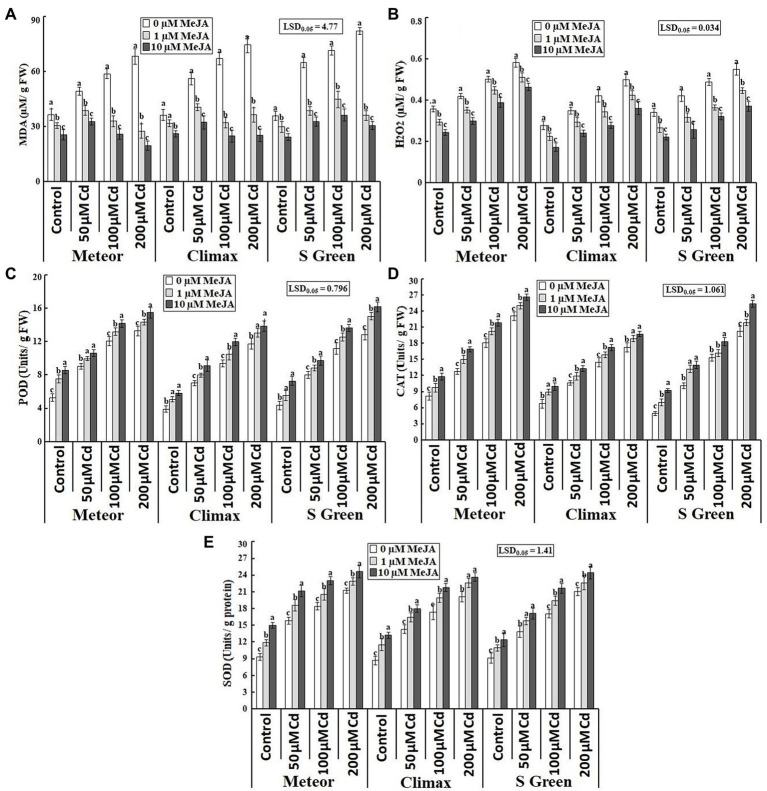
Effect of CdCl2 (50, 100, and 200 μm) and MeJA (1 and 10 μm) on **(A)** Lipid peroxidation; **(B)** ROS; **(C)** POD; **(D)** CAT; and **(E)** SOD of three pea cultivars. Bars show a mean of 3 three replicates ± standard deviation. Letters a, b, and c showed significant differences between means of different treatments.

Cadmium toxicity considerably affected lipid peroxidation by increasing MDA and H_2_O_2_ contents in all Cd-stressed pea plants that were substantially reduced through the treatment of MeJA in typical and Cd-stressed plants under investigation (Table 1; Figures 4A,B). The exogenous application of 10 μm MeJA showed a more significant reduction of MDA and H_2_O_2_ contents at all cadmium concentrations in a cultivar-dependent way. For example, at 200 μm CdCl_2_ stress, a considerable decrease in MDA and H_2_O_2_ contents was observed in Meteor (71.26%) and S-green (32.7%), respectively.

Cadmium stress also induced considerable changes in the activities of antioxidative enzymes among all pea cultivars, including POD, CAT, and SOD. The activities of all these enzymes increased significantly with increasing cadmium concentration; however, much increase was observed in Cd + MeJA treatment (Table 1; Figures 4C–E). The maximum increase in activities of all these enzymes was observed at 200 μm CdCl_2_ plus 10 μm MeJA in all cultivars, particularly POD and CAT activity in S-green by 26.04 and 25.63%, respectively, while SOD activity in Climax by 17.3%.

## Discussion

Plants are susceptible to heavy metal toxicity, including Cd. Cd inhibits plant growth *via* interference with cell division, hormonal homeostasis, photosynthesis, nutrient uptake, and enhancing oxidative stress (Rizwan et al., 2018; Huang et al., 2020; Dobrikova et al., 2021). In this study, the exposure of Cd considerably repressed the growth of pea plants, which is similar to earlier studies with different crop species (Huang et al., 2020; Shahzad et al., 2021; Wei et al., 2021). However, the toxic effects were compensated by the addition of MeJA. Previous research has shown that MeJA can modulate growth by altering antioxidant enzymatic activity increasing photosynthetic pigments and hormones under both biotic and abiotic stresses(Yan et al., 2015; Butt et al., 2019; Tayyab et al., 2020). In view of these reports, it is suggested that the ameliorative effect of MeJA on the growth of pea cultivars under Cd stress was possibly due to its impact on photosynthetic pigments, chloroplastic activity, and antioxidant enzymes.

Plant growth suppression caused by Cd is linked with changes in photosynthetic pigments, chloroplastic structures, and photosynthetic rate (Per et al., 2016; Qiu et al., 2020). In this study, Cd stress reduced the chl a, chl b, total chl, and carotenoids in pea leaves. The decrease in photosynthetic pigment content in three pea cultivars can be explained in view of some earlier studies in which it has been demonstrated that the high accumulation of Cd in leaves altered chlorophyll metabolism by affecting the activity of chlorophyll biosynthesis enzymes and chloroplast ultrastructure (Qiu et al., 2020). Application of MeJA increased photosynthetic pigments in pea cultivars. These results are analogous to earlier studies that demonstrated that MeJA promoted the accumulation of photosynthetic pigments in citrus (Qiu et al., 2020) and *Vicia faba* (Ahmad et al., 2017) exposed to metal stress.

The photosynthetic efficiency of plants is critical because it directly affects plant growth and productivity (Athar and Ashraf, 2005; Ogbaga et al., 2018). Cd stress significantly affected PSII structural and functional activity in all pea cultivars, which is in agreement with earlier research in which it was found that higher accumulation of Cd reduces the PSII structural ability and functional activity of Thellungiella salsuginea plants (Goussi et al., 2018). They explained it as Cd stress damages the PSII antenna and core resulting in reduced efficiency of PSII and impaired electron transport, as has been observed in this study. However, MeJA treatment enhanced the quantum yield [Y(II) and (Fv/Fm)] and electron transport rate ETR (II) of PSII. Several previous reports showed that MeJA improved photosynthesis-related attributes in Mentha arvensis (Zaid and Mohammad, 2018) and *Triticum aestivum* (Kaya et al., 2021) MeJA improved the parameters mentioned above.

On the other hand, increased energy dissipation in all Cd-stressed pea plants, as evidenced by elevated Y(NPQ) and Y(NO) values, indicates more energy loss along with reduced efficiency of PSII. This could be due to a reduction in carbon dioxide fixation in the Calvin cycle. Due to Cd stress, the production of excessive ROS denatures the D1 protein of PSII (Ruban et al., 2012). Usually, non-photochemical quenching gives a quick response and prevents ROS generation by dissipation of light energy in the form of heat through the antenna complex (Lambrev et al., 2012).

In plants, heavy metal toxicity usually causes oxidative stress due to excessive ROS production, including O^2−^, H_2_O_2_, and ^·^OH. These compounds are highly reactive and toxic; they cause membrane damage and oxidize macromolecules like carbohydrates, proteins, lipids, and DNA (Gill and Tuteja, 2010). Cellular damage caused by heavy metal stress results in the increased production of H_2_O_2_ that ultimately enhances the peroxidation of membrane fatty acids leading to increased MDA content. MDA is commonly used for plants as the marker for lipid damage and lipid peroxidation that disrupts membrane fluidity, enhances electrolyte leakage, inhibits enzymes’ activity, and interferes with protein channeling (Garg and Manchanda, 2009). In the present study, exposure of pea plants with Cd stress increased the H_2_O_2_ and MDA content. These results were similar to many studies conducted on *P. sativum* (Romero-Puertas et al., 2004) and Vigna radiata (Ahmad et al., 2011). The supplementation of MeJA to Cd-stressed pea plants significantly reduced the H_2_O_2_ and MDA levels indicating that MeJA has the potential to overcome oxidative stress found in intracellular membranes and cell membranes of all pea cultivars. A study supporting this conclusion showed that the application of MeJA enhanced the enzyme activities (APX, CAT, and SOD) of Bunium persicum plants exposed to Cd stress. These results were further supported by Chen et al. (2014), who demonstrated that supplementation of JA reduced MDA concentration in Kandelia obovate in response to Cd stress.

As discussed above, antioxidative machinery can scavenge ROS and protect plants against oxidative damage. In the present study, the increased antioxidant enzyme activities (CAT, POD, and SOD) correspond to those levels observed in *B. juncea* (Ahmad et al., 2016), Solanum lycopersicum (Cherif et al., 2011), and *P. vulgaris* (Rady, 2011). Moreover, in Capsicum frutescens plants, JA supplementation to Cd-stressed seedlings increased antioxidative enzyme activities and chlorophyll production (Yan et al., 2015). The application of JA also improved tolerance against Cd stress by increasing phytochelatin levels and activating defense-related genes (Maksymiec et al., 2007). Enzymatic activities enhanced by the JA supplementation can be attributed to the direct association with radicals, such as superoxide. It might be due to cells’ improved ROS quenching capability by producing antioxidative enzymes (Hsu and Kao, 2007; Sirhindi et al., 2016). Overall, MeJA-mediated improvement against Cd toxicity in lipid membranes appears to be linked to increased antioxidant ability.

Furthermore, cadmium present in nutrient solution and soil can be easily absorbed by plants’ roots and transferred to other plant tissues. The highest Cd content in leaves was found in this investigation, which was remarkably lowered by MeJA treatment. Heavy metals usually compete with Zn^2+^, Ca^2+^, Fe^2+^, Mg^2+^, and Mn^2+^ cations to access root cells through these cations transporters. It can be assumed that MeJA treatment enhanced the physiological parameters of all pea varieties and increased the uptake of valuable cations, decreasing Cd content and Cd^2+^ influxes in pea plants, similar to a previous study conducted on tomato plants (Wei et al., 2021). JA application reduced the uptake of Cd and improved antioxidant machinery in Kandelia obovata (Chen et al., 2014). However, the underlying mechanisms concerning how MeJA lowers the uptake of Cd are still unknown.

## Conclusion

In the present study, Cd stress caused the higher Cd accumulation in pea plants, disturbing the metabolism for photosynthetic pigment biosynthesis and damaged photosynthetic machinery. Application of MeJA reduced the toxic effects of Cd on photosynthetic pigments and maintained the PSII activity by lowering uptake of Cd, downregulation of electron transport from PSII to PSI, increase in the photoprotective component of NPQ, and partial increase in activities of antioxidant enzymes. This helped the plants maintain cellular redox balance, thereby resulting in lower membrane damage. Thus, it can be inferred that the MeJA-induced growth improvement is related to (i) reduced Cd uptake by roots, (ii) optimization of the photosynthetic apparatus or photosynthetic rate, and (iii) enhanced antioxidant activity.

## Data Availability Statement

The datasets presented in this study can be found in online repositories. The names of the repository/repositories and accession number(s) can be found in the article/supplementary material.

## Author Contributions

SR: conceptualization and supervision. SR and HM: methodology. SR, H-u-RA, and SN: validation. MR and ZZ: formal analysis. Mehwish and SB: investigation. SR: resources. Mehwish: data curation. SB: writing—original draft preparation. MS, MB, WS, SR, HM, H-u-RA, MR and AE: writing—review and editing. SN and MR: visualization. HM: project administration. CCO: writing—review and editing. All authors contributed to the article and approved the submitted version.

## Conflict of Interest

The authors declare that the research was conducted in the absence of any commercial or financial relationships that could be construed as a potential conflict of interest.

## Publisher’s Note

All claims expressed in this article are solely those of the authors and do not necessarily represent those of their affiliated organizations, or those of the publisher, the editors and the reviewers. Any product that may be evaluated in this article, or claim that may be made by its manufacturer, is not guaranteed or endorsed by the publisher.
